# When did you stop speaking to yourself? Age-related differences in adolescents’ world knowledge-based audience design

**DOI:** 10.1098/rsos.220305

**Published:** 2022-11-30

**Authors:** Caroline Arvidsson, David Pagmar, Julia Uddén

**Affiliations:** ^1^ Department of Linguistics, Stockholm University, Stockholm, Sweden; ^2^ Department of Psychology, Stockholm University, Stockholm, Sweden

**Keywords:** audience design, pragmatic development, referential production, adolescence, theory of mind, executive functions

## Abstract

The ability to adapt utterances to the world knowledge of one’s addressee is undeniably ubiquitous in human social cognition, but its development and association with other cognitive mechanisms during adolescence have not been studied. In an online production task, we measured the ability of children entering adolescence (ages 11–12, *M* = 11.8, N=29, 17 girls) and adolescents (ages 15–16, *M* = 15.9, N=29, 17 girls) to tailor referential expressions in accordance with the inferred world knowledge of their addressee—an ability we refer to as world knowledge-based audience design (AD). A post-test survey showed that both age groups held similar assumptions about the addressees’ knowledge of referents, but the younger age group did not consistently adapt their utterances in accordance with these assumptions during online production, resulting in a significantly improved AD behaviour across age groups. We also investigated the reliance of AD on executive functions (EF). Executive functioning (as reflected by performance on the Wisconsin card sorting task) increased significantly with age, but did not explain the age-related increase in AD performance. We thus provide evidence in support of an adolescent development of world knowledge-based AD over and above development of EF.

## 1. Introduction

When describing the general conversational conduct of children, Piaget stated that children under the age of 7 virtually speak to themselves [1]. This can be seen as an early observation that young children do not engage in what is frequently termed *audience design* (AD) [2] to the same extent as adults [3–5]. AD entails adjusting utterances to suit the needs of interlocutors. This listener-catering behaviour operates on all levels of language, but is particularly salient in referential production, in which speakers often need to take into account the *world knowledge* of their interlocutor. For example, consider a situation in which a pragmatically skilled speaker wishes to refer to her favourite computer game. If computer games is not assumed to be an area of knowledge for the addressee (e.g. her 90-year-old grandmother), the speaker will be inclined to produce a more informative noun phrase (e.g. the game that mom bought me for my birthday) instead of a proper name (e.g. Minecraft) to maximize the possibility of overlap between her and her interlocutors’ mental representation as to what is being denoted. From a Gricean perspective, engaging in AD entails adhering to the Gricean maxim of quantity [6], by making one’s conversational contribution adequately informative for a particular addressee.

Our previous investigations [3,5] suggest that 7-year-olds do not adhere to the maxim of quantity by tailoring utterances to the inferred world knowledge of addressees, to the same extent as adults. We are interested in the adolescent period as variation in this ability, since it might explain the substantial individual differences in the same ability that we observed in young adults [5]. In other words, we hypothesize that this ability develops in adolescence—a transitional period during which an individual’s social world becomes increasingly peer-oriented and complex [7]. The developmental trajectory of pragmatic abilities in the adolescent population is also interesting because adolescence is qualitatively distinct from childhood and adulthood, characterized by its own unique set of linguistic and sociocognitive goals [7–10]. We are the first to investigate age-related differences in the ability to adapt utterances to the assumed world knowledge of addressees in adolescence, and thereby contribute to the understanding of how AD, and more generally, pragmatic ability, develops throughout the lifespan.

Our approach is built on the processing perspective prominent in Clark [11]. From the processing perspective, having assumptions about others’ knowledge states can be distinguished from adapting one’s utterances in accordance with these assumptions during online communication. In what follows, we use *assumed knowledge* to denote the speaker’s assumption regarding the addressee’s knowledge state. The term AD, on the other hand, stands for the process in which speakers adapt their utterances according to these assumptions. In the current study, we investigate age-related differences with respect to AD in middle childhood (11–12) and adolescence (15–16) by utilization of a task design that distinguishes between these aspects. We present a novel AD task and scoring method that requires speakers to use the assumed world knowledge of interlocutors during online referential production.

An important input to the process of tailoring utterances to the assumed knowledge state of one’s interlocutor (i.e. the AD process) is the discernment between what is common ground (knowledge shared by both interlocutors) [2], and what is privileged ground (knowledge exclusively available to the speaker) [12]. In the following sections, the development of using common ground information during online communication and its potential cognitive underpinnings are reviewed.

### 1.1. Development of common ground use during online communication

Viewing common ground as the intersection of the speaker’s and the addressee’s area of knowledge with respect to a given referent, the speaker’s process of finding common ground could be construed as including a step where aspects of *theory of mind* (ToM) are used. ToM is known as a collection of concepts that inter alia allow individuals to perceive others as mental beings driven by their own knowledge state [13,14]. As ToM is an ability with many facets, we will focus on aspects needed for consistent production of adequately informative utterances. One plausible prerequisite is the insight that others’ knowledge states can differ from one’s own. Children exhibit this ability from a young age [15]. For example, in classical false-belief tasks, 3- to 4-year-olds are able to keep track of and reproduce specific information that a play-partner has not seen due to being absent at the time when the information was provided [16]. Previous studies indicate that there is a link between performance on various ToM tasks, including false-belief tasks, and the ability to use common ground information during referential comprehension and production in early and middle childhood (ages 3–10) [17–19]. However, 4- to 6-year-olds do not use information regarding the visual perspective of interlocutors during online production to the same extent as adults [4,20]. This suggests that while the assumptions about others’ knowledge as such may be present and accessible for children, they are not always used during online production. Such a pattern was observed in a study on 10-year-olds [21], showing that while children are able to find common ground with respect to what has been mentioned in the previous linguistic context, they do not consistently deploy this common ground information during production. Findings from comprehension studies suggest that the ability to use common ground online develops much later. For example, one study [22] showed that the ability to use information regarding the speaker’s perspective in order to correctly choose a target referent develops throughout adolescence (ages 11–18). This suggests that the use of common ground information during online communication continues to develop well beyond the pre-school years. Indeed, studies on socio-cognitive development indicate that adolescence is a crucial period for developing ToM abilities [23,24]. In addition, the ability to engage in AD may depend on which kind of information is used online (for example, visual perspective or world knowledge).

### 1.2. Development of AD during online referential production

Because of its universality and simplicity, referential production has gained substantial attention within AD research [25,26]. Traditional referential communication tasks aim to measure the ability to adapt utterances to the knowledge state of addressees, typically by modifying interlocutors’ visual access to referents. An example of such a task is the well-established director task [27]. Performance on the director task (specifically in the ‘common ground condition’) is typically assessed by counting utterances in which participants use disambiguating adjectives to guide an addressee in choosing one specific object among multiple competing objects displayed [4]. Children are less effective than adults at making their referential expressions as informative as required in these types of tasks. For example, in only 39% of these trials, 4- to 5-year-olds provided disambiguating adjectives [20], while 5- to 6-year-old children used disambiguating adjectives in 75% of trials [4], and adults provided disambiguating adjectives in 100% of trials [4]. This suggests that AD develops successively during childhood, and that children reaching school age have not yet developed into adult-level communicators. It is, however, important to keep in mind that the adult population exhibits variation with respect to communication skills [5,28–31] so that individual variability can trump the variability between the child, adolescent and adult populations—meaning that e.g. the best children/adolescents may be more proficient than the worst adult individuals.

Less is known regarding the development of AD in adolescence. However, we recently conducted an AD task with children, showing that 7-year-olds fail to adapt their utterances to addressee’s world knowledge in more than half of trials [3]. The children’s AD performance corresponded to the performance of the low pragmatic ability group in another of our lab’s studies, using a similar AD task, but with adults [5]. Hence, we expected to see an increase in this ability throughout adolescence. Moreover, Fukumura [32] found that 11- to 16-year-old participants provided disambiguating adjectives when necessary from the addressee’s perspective nearly as often as adults, but failed to omit size-adjectives when redundant. Albeit these findings may reflect a true late and successive development of the ability to take information concerning the listener’s visual perspective into account, AD development in adolescence was not the focus of the study, i.e. variation within 11- to 16-year-olds was not investigated. In addition, utterance adaptation to the visual perspective of interlocutors is arguably significantly less prevalent in real-life conversation than adaptation to the assumed world knowledge of interlocutors. We wanted to improve the state of this literature along these lines, as the director task has been criticized for not necessarily tapping ToM, but other domain-general cognitive functions such as selective visual attention [33].

In the current study, we investigated age-related differences in AD during referential production, using an innovative task design [3,5], that required speakers to engage in AD by considering the world knowledge of interlocutors, rather than their visual access to referents. We compared performance on this task in two age groups: children entering adolescence (11–12) and middle adolescents (15–16). While views regarding at which age adolescence is reached vary across cultures, most researchers agree that its commencement coincides with the onset of puberty [7]. We hypothesized that the ability to adapt utterances in accordance with others’ assumed world knowledge, and thus the ability to engage in world knowledge-based AD, develops in these age-ranges—a prediction consistent with the developmental literature on common ground use during online communication (see §1.1). Additional support for this hypothesis was the observation that adults’ perspective taking during referential production is subserved by medial prefrontal and temporo-parietal regions [34]—regions that undergo structural and functional changes during adolescence [7]. Crucially, an age-related increase in world knowledge-based AD in the investigated age groups could reflect an age-related difference with regards to the assumptions held about others’ world knowledge as such, or a development in the ability to adapt their utterances according to these assumptions online, and we, therefore, addressed this issue experimentally (see §2.3.2). As AD has been hypothesized to rely on more domain-general executive functions (EF) [35], we now turn to this possibility.

### 1.3. The role of executive functions in AD

Recall that young children show awareness of others’ knowledge states [16], but fail to consistently use this information during conversation [3,4,20]. Building on this literature, it was hypothesized that the usage of common ground information during online communication relies on the development of EF [20]. Again, taking the processing perspective, the term EF denotes a set of domain-general, higher order processes that facilitate flexible and goal-oriented behaviour [36]. It has been proposed that EF play a crucial role in finding common ground during online communication by (i) suppressing an initial—egocentric—perspective, (ii) shifting to a listener-friendly perspective and (iii) maintaining and updating contextual, social and linguistic information relevant to one’s conversational contribution [35]. Roughly, these suggestions match the three well-studied components of EF: inhibition, cognitive flexibility and working memory [37–39]. In an extensive review [40], it was concluded that while EF usually correlate with global measures of pragmatic ability, the relation between EF and specific pragmatic abilities remains unsettled.

Developmentally, no link between EF (working memory, inhibition and cognitive flexibility) and the ability to provide sufficiently detailed referential expressions was observed when younger children participated in a visual perspective-taking task (years: 4–5) [20], whereas a link between EF subfunctions (cognitive flexibility, working memory) and children’s ability to successfully produce sufficiently detailed, as well as repair insufficient referential expressions, has repeatedly been observed in 4- to 6-year-olds [41,42]. On the adult side, the literature is also mixed, but a recent large-scale study found no correlation between world knowledge-based AD and working memory capacity in adults (see [5] and references therein).

In order to investigate whether a potential increase of AD across age groups reflects a development of processes special to the communicative situation rather than general EF, we measured EF using the Wisconsin card sorting task (WCST) (see further §2.3.3.). Due to mixed evidence, we hypothesized that although some of the potentially observed AD development might be explained by adolescents’ EF, AD development would also manifest over and above EF development.

### 1.4. Summary of the approach

In order to answer the questions regarding the development of world knowledge-based AD in adolescence, we conducted a novel version of the director task, requiring speakers to use common ground information online, by considering the world knowledge, rather than visual perspective, of addressees. We expected a significant age-related difference in AD performance as reflected by a greater propensity in the older adolescents to adapt utterances to the assumed knowledge of their addressee’s, even when controlling for a probable age-related difference in the assumptions held, and EF.

## 2. Methods

### 2.1. Participants

The sample consisted of 58 adolescents from two age groups (11–12 years: *N* = 29, 17 girls, *M*_age_ = 11.9; 15–16 years: *N* = 29, 17 girls, *M*_age_ = 15.8), recruited from two schools in Värmdö municipality, Stockholm County. The two participating schools (one middle school and one high school) by which information about the study was disseminated, were chosen due to their close proximity; many of the pupils attending the participating high school had previously attended the participating middle school.

The study was approved by the Swedish Ethical Review Authority (Dnr 2020-07083). Informed consent was obtained from all participants and the legal guardian(s) of participants in the younger age group. Participants received small tokens of appreciation (value less than or equal to 50 SEK) upon participation. Participants were (i) fluent speakers of Swedish and (ii) had not been diagnosed with any language impairment, as ascertained by a parental-report (younger age group) or self-report (older age group) questionnaire. Three participants were excluded prior to analysis due to reports of having been diagnosed with ASD, ADHD and/or DLD. One participant was excluded after having more than half of trials deleted during the analysis of a post-test survey (§2.3.2) that controlled for knowledge of referents and assumption about listeners’ knowledge of stimuli (see further §2.4.1).

### 2.2. Materials and procedure

During one full half-hour session, the participant was presented with two tasks, respectively, designed to measure (i) AD in referential production and (ii) EF.

### 2.3. Tasks

#### 2.3.1. Test of audience design

**Figure 1.**
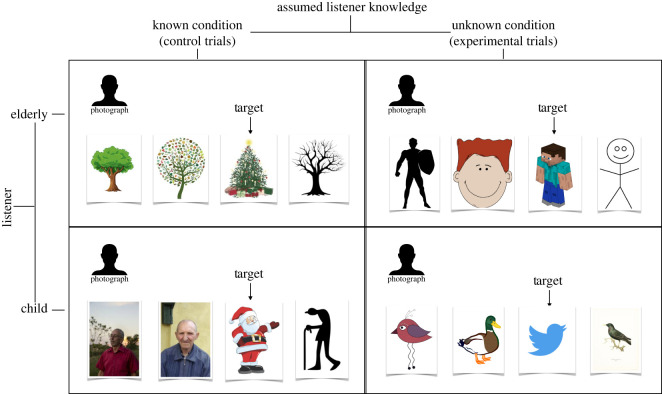
Example of four trials showing the AD task’s two conditions. In the upper corner was a photograph of the listener. Target referents in known condition: Christmas tree, Santa Claus. Targets in unknown condition: Minecraft character, the Twitter logo. Images are in public domain (CC0 1.0).

The referential production task was modified from two prior studies [3,5]. Its aim was to measure adolescents’ ability to adapt their referential expressions to the inferred world knowledge of their interlocutor. In each trial, a picture of the addressee (either a child or an elderly woman) was presented in the upper corner of a laptop screen. We used one picture per addressee throughout the trials. Participants were instructed to record a short message that would enable their addressee to choose the target referent. Successful referential production entailed describing the target referent, i.e. providing at least one visual feature of the target referent by for example uttering a modified noun phrase (see table 1), rather than simply naming the referent, when the participant assumed that the listener did not know the name of the target referent. At the bottom of the screen, one target referent (e.g. a video game character, a musician or other individual/object) and three competitor referents were displayed. In both of the task’s conditions, the participant knew the name of the target referent but was expected to assume that the addressee either (i) did not know the name of the target (unknown test condition) or (ii) did know the name of the target (known control condition). An example of four trials from the task’s two conditions is given in figure 1. Participants’ knowledge of each target referent and their assumptions regarding the addressee’s knowledge of each target referent were controlled for through a post-test survey (see §2.3.2).

**Table 1.** Translated examples (from Swedish) of descriptions of target referents. To provide referential expressions that successfully distinguished targets from competitors, a noun phrase with a maximum of one modifier (an adjectival or prepositional phrase) was necessary. A table listing all target referents and example descriptions is available in electronic supplementary material, table S1.

target	competitors	example of target description
Christmas tree	trees	‘The tree with a star’
Santa Claus	elderly males without beards	‘The bearded man’
Minecraft character^a^	cartoon figures (none in t-shirt)	‘The man in t-shirt’
Twitter logo	pink, white or greenish birds	‘The blue bird’

^a^The experimenter learned from the participants that the name of this character is actually ‘Steve’.

Prior to participation, the participant was instructed on the age and general interests of the addressees (edlerly woman: 90 years old, knitting; child: 4 years old, playing at kindergarden). The task had one initial practice trial in which the participant was instructed to produce an utterance that would guide their addressee to choose the target picture. Because pilot runs of the experiment showed that participants that were presented with an unknown condition trial in the practice trial described referents throughout the entire experiment (irrespective of trial condition), the practice trial was always a known condition trial (in which referring by describing was redundant) in order to avoid that participants had misunderstood the task by thinking that they were supposed to consistently describe target referents. Thus, if the participant referred to the target in the practice trial as ‘The tree with a star’, the experimenter always said ‘Sure, but you could also just say Christmas tree, right?’.

To control for processing efforts of maintaining disambiguating features of competing referents in memory, the competitor stimuli were chosen so that a noun phrase containing no more than one modifier (e.g. an adjectival or a prepositional phrase) was enough to comprise an unambiguous target description (see table 1). Furthermore, the selected stimuli were adapted so that target referents would be known to adolescents, and also could be assumed by adolescents to be either unknown or known to specific addressees, depending on the listener’s age and gender. This was verified through an online questionnaire with six respondents (age 11) that were not part of the experiment.

Building on results from 7-year-olds [3], we assumed that participants would name target referents in the known trials and that a relatively small number of trials would suffice to record that behaviour. We, however, expected that pragmatic development (taking place between age 7 through the investigated age groups in the present study) would be observed as variability in the unknown trials, i.e. that not all participants would show the more pragmatically developed behaviour of describing referents based on assumed lack of listener knowledge. For this reason, we doubled the number of trials in the unknown condition. Altogether, the task consisted of 36 trials (24 in the unknown condition, 12 in the known condition). Four randomized versions of the experiment were presented in an evenly balanced fashion across participants. The test was implemented using PsychoPy [43].

Scoring the AD task, we wanted to (i) get an overall estimate of how inclined the participant was to flexibly switch referring strategy (describing/naming) in accordance with their assumptions about the addressee’s knowledge state and (ii) account for the possibility that the participant solely used describing as an overall strategy. Thus, individual performance on the AD task was computed by subtracting the proportion of sufficient (i.e. describing) referential expressions in the unknown (experimental) condition from the proportion of redundant (i.e. describing) referential expressions in the known (control) condition. This resulted in an AD score somewhere between −1.0 (participants who only described when describing was redundant) and 1.0 (participants who only described when describing was necessary).

#### 2.3.2. AD post-test survey: controlling for assumed knowledge

We conducted a post-test survey to control for the participant’s assumptions regarding the addressees’ knowledge of the target referents. The participant was presented with pictures of each target referent from the AD task and furthermore answered whether they believed that the addressee (the child or the elderly woman) knew the name of the referent. Trials in which the participant’s assumptions matched the conditions (i.e. unknown condition: assumed that the addressee did not know the name of the referent; known condition: assumed that the addressee knew the name of the referent) were labelled matching assumption trials (similarly for the non-matching assumption trials).

Without this further testing, performance on the AD task could either reflect the ability to adapt utterances to the assumed knowledge of one’s addressees, or one’s assumptions as such. For example, in the unknown condition, the participant could name the referent (i) because they assumed that the addressee knew the name of the object or (ii) in spite of assuming that the addressee did not know the name of the referent. Our aim was to distinguish between these two cases, as (ii) could be considered an AD failure, while (i) could not.

Furthermore, based on this data, we computed a knowledge attribution score (KAS). KAS was defined as the number of matching assumption trials, divided by the total number of trials. This resulted in a value between 0.0 and 1.0. KAS reflects the presence or absence of the specific representations that need to be used online to succeed in the AD task. We included the KAS score in the model to explicitly test for the possibility that age-related differences in AD may be related to age-related differences in forming typical assumptions about other’s knowledge states as such.

#### 2.3.3. Test of executive functions

To assess adolescents’ EF, a computerized version of the WCST was administered. The WCST is one of the most frequently used neuropsychological measures of EF [44–47]. Participants matched cards according to a hidden matching rule (colour, number or shape) that changed implicitly after 10 consecutive trials. The task required participants to figure out the rule by trial and error with feedback responses after each matching. Two WCST scores were used: proportion *perseverative errors* and proportion *non-perseverative errors*. A perseverative error was defined as a failure to switch matching rule after a set shift. A non-perseverative error was defined as any error that was not perseverative. Both perseverative errors and non-perseverative errors were predicted by measures of cognitive flexibility and working memory capacity in adolescence [48,49]. Conceptually, a perseverative error represents inability to flexibly shift matching-strategy when required and a non-perseverative error represents inability to maintain matching-strategy when required [50]. The computerized implementation of WCST used in the current investigation consisted of 72 trials (including 12 practice trials) and was available at PsyToolkit’s [51,52] experiment library.^1^

### 2.4. Analysis

#### 2.4.1. Data processing

As planned, we removed data from (i) trials in which the participant did not know the name of the target referent (6% of trials) and (ii) non-matching assumption trials (5% of trials). One participant was excluded from the analysis, since as many as 17 trials (12 non-matching assumption trials) were removed from this particular participant. The remaining participants (*N* = 58) had a minimum of 13 trials (*M* = 20.44) in the unknown condition and a minimum of 10 trials (*M* = 11.68) in the known condition (starting number of trials: 24 unknown condition, 12 known condition).

A Shapiro–Wilks normality test showed that AD data were positively skewed (data were normally distributed for the younger age group, whereas 66% of the older adolescents had a score of 0.7 or higher, with the maximum and minimum possible scores being 1.0 and −1.0). Thus, data were transformed using a rank-based inverse normal (RIN) transformation, which, when compared to a wide range of transformation methods, have been shown to be most beneficial with respect to statistical power on moderate sample sizes (*N* ≥ 20) [53]. All data and scripts are available at [54].

#### 2.4.2. Statistical analyses

An *a priori* statistical power analysis was performed for sample size estimation, based on data from the published study by Fukumura [32]. The study inter alia involved measuring the ability to adapt referential expressions to listeners’ visual access to objects in TD children (*N* = 20, aged 6–10) and TD adolescents (*N* = 20, aged 11–16). Effect size (Cohen’s *d*) in regard to difference in performance between these two age groups was 1.9. With an alpha = 0.05 and power = 0.80, the projected sample size needed for a regression model with this effect size and four independent variables is approximately *N* = 39. This suggests that our sample size of 58 is more than adequate for the main objective of this study, which shares similarities with [32] with respect to the measured phenomenon and investigated populations. The power analysis was performed using the software GPower [55].

One frequentist univariate generalized linear model (GLM) (Model 1a) and one corresponding Bayesian GLM (Model 1b) were used to test AD as a function of (i) age, (ii) EF and (iii) assumed knowledge. In Model 1a and Model 1b, AD score was dependent variable and the independent variable testing for (i) was AGE GROUP (11–12 versus 15–16), while perseverative errors and non-perseverative errors tested for (ii) and KAS tested for (iii). The continuous predictors in these models were centred (i.e. subtracted by their sample means to produce revised sample means of zero) upon model fitting. This is often recommended in order to reduce multicollinearity between predictors and the interaction terms created from them [56–58]. Multivariate linear regression was conducted to investigate whether EF and assumed knowledge depended on age group (Model 2). In this model, AGE GROUP was the independent variable, and KAS, perseverative errors and non-perseverative errors were dependent variables. *p*-values were adjusted for multiple comparisons (Bonferroni correction). Furthermore, to account for the possibility that the role of EF in referential production varies across ages, as discussed in [40], we performed an additional test (Model 3) which included the main effects in Model 1a, but also the interaction AGE GROUP * Perseverative errors. The results and power analysis for Model 3 are provided in electronic supplementary material, table S2. Note that the presence or absence of interaction terms in the models did not change the results in any notable way.

Statistical modelling was performed with the statistical software R [59]. Model 1a and 3 were fit using the built-in function glm with an alpha level of *α* = 0.05. Model 1b was fit using package rstanarm (function stan_glm) [60]. Package bayestestR (function bayesfactor_parameters) [61] was used to calculate Bayes factor_10_ (BF_10_) with default priors. BF_10_ was interpreted in accordance with Lee and Wagenmaker’s classification categories [62]. Predictors in Model 1a and 1b were centred using R’s built-in function scale. The multivariate linear regression model (Model 2) was built with the function lm. The *p*-values in Model 2 were adjusted for multiple comparisons using the function p.adjust. We calculated variance inflation factor (VIF) to confirm that the independent variables did not exhibit a problematic amount of collinearity. VIF values greater than 5 are assumed to indicate a problematic amount of collinearity [63]. VIFs for all our variables were less than 1.7. We included a correlation matrix for all our continuous variables in electronic supplementary material, table S1. The correlation was computed using the chart.correlation function with Spearman’s rank-order correlation as the chosen method in the PerformanceAnalytics package [64].

## 3. Results

**Figure 2.**
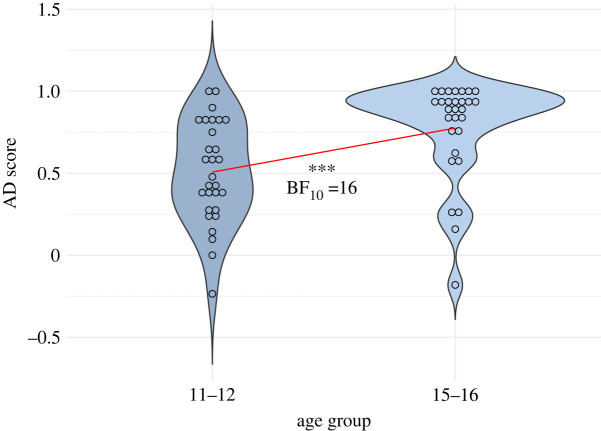
Non-transformed individual AD score by age group. The red line indicates the age-related difference in mean ability to adapt utterances to the assumed frame of reference of the addressee.

The older age group (15–16) scored higher and made fewer errors than the younger age group (11–12) on all tasks (see table 2 and figure 2). The older age group also exhibited less variation in task performance. Table 2 shows descriptive statistics for all test scores, grouped by age.

**Table 2.** Descriptive statistics for all test scores (non-transformed and non-centred), grouped by age.

	AD score		perseverative errors		non-perseverative errors		KAS	
age group	11–12	15–16	11–12	15–16	11–12	15–16	11–12	15–16
min	−0.24	−0.18	0.08	0.08	0.03	0.02	0.71	0.83
max	1.00	1.00	0.34	0.31	0.30	0.13	1.00	1.00
mean	0.51	0.78	0.18	0.14	0.12	0.07	0.94	0.96
s.d.	0.31	0.30	0.06	0.05	0.07	0.03	0.07	0.05

### 3.1. Audience design as a function of age, EF and assumed knowledge (Model 1a and 1b)

Compared to their younger peers, 15- to 16-year-olds described referents they assumed to be unknown to addressees more often (in 93% versus 83% of unknown condition trials), and described referents they assumed to be known to their addressees less often (in 16% versus 30% of known condition trials). Figure 2 shows individual AD score by age group.

We performed one frequentist (Model 1a) and one Bayesian (Model 1b) GLM with AD as dependent variable and AGE GROUP, perseverative errors, non-perseverative errors and KAS as independent variables. In Model 1a, AGE GROUP was significant *T*_53_ = 3.72, *p* < 0.001. No other terms were significant (perseverative errors: *T*_53_ = 1.16, *p* = 0.25; non-perseverative errors: *T*_53_ = 0.39, *p* = 0.70; KAS: 0.69, *p* = 0.49). Model 1b found strong positive evidence in favour of AGE GROUP (BF_10_ = 16.30), but found moderate negative evidence against perseverative errors (BF_10_ = 1/15.21) and strong negative evidence against non-perseverative errors (BF_10_ = 1/8.46) and KAS (BF_10_ = 1/14.14).

### 3.2. Executive functions and assumed knowledge as a function of age (Model 2)

The multivariate linear model with AGE GROUP as independent variable, and perseverative errors, non-perseverative errors and KAS as dependent variables showed the following: AGE GROUP was significant for both the EF scores (perseverative errors: *F*_1,54_ = 1.71, *p* < 0.05; non-perseverative errors: *F*_1,54_ = 9.55, *p* < 0.01), but not for KAS (*F*_1,54_ = 1.3, *p* = 0.8). All *p*-values in Model 2 were adjusted for multiple comparisons.

## 4. Discussion

Using referential production as a test bed, we have shown significant age-related differences in AD between children entering adolescence (11–12) and middle adolescents (15–16). Our results show that children entering adolescence (11–12) do not adapt utterances in accordance with their own assumptions about their addressees’ knowledge state during online production to the same extent as middle adolescents (15–16), see figure 2.

**Figure 3.**
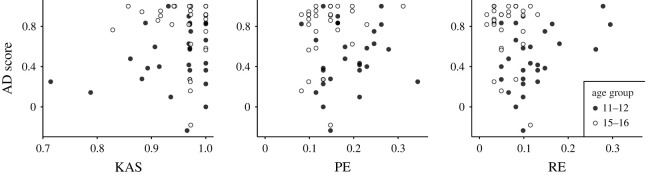
Relation between AD performance (non-transformed scores) and KAS (knowledge attribution score; leftmost panel), perseverative errors (PE; indicating ability to flexibly switch matching rule after set-shift in the WCST; middle panel) and non-perseverative errors (NE; indicating ability to maintain a correct matching behaviour during a set in the WCST; rightmost panel). Data points represent individual performance (non-centred) and are colour-coded by age group.

We provide evidence that this difference cannot be explained by measures of their ability to form typical assumptions regarding others’ knowledge states (as there was no such relationship, see figure 3). Nor were any age-related differences in the assumed knowledge as such observed (Bayes factor showed strong evidence against such an effect). In other words, the relevant assumptions were already present and ready to be accessed in early adolescence, but only when they were specifically prompted for this information. Furthermore, an expected age-related difference in EF was observed across groups but there was no link between AD and EF, see figure 3. We thus provide evidence suggesting that 11- to 12- and 15- to 16-year-olds’ ability to engage in AD with respect to adapting utterances to the inferred world knowledge of their addressees cannot be explained by measures of their EF.

Our findings support accounts from referential comprehension, suggesting that adolescence is a period of significant growth with respect to the ability to use common ground information during online communication [22]. While 7-year-old children fail to flexibly adapt their utterances to others' assumed world knowledge [3], adolescence appears to be a sensitive period in this respect. Indeed, since the KAS score was close to the maximum possible score on a group level, the ability to form typical assumptions about others’ world knowledge develops first, and then, interestingly, it takes a while before the ability to use these representations online emerges. This may be ascribed to the functional reorganizations and structural changes of brain regions important to perspective taking during online communication that take place in adolescence [7,34,65], and supports a processing model distinguishing between the mental representations of others’ knowledge as such and the usage of these representations during online communication [35].

We present a novel AD paradigm that requires speakers to consider the world knowledge of interlocutors, by taking into account specific traits of their addressee (in this case, age). The identity of the addressee has for decades been described as one of the main sources for decoding what is in common ground (see for example Clark and Murphy’s [2] seminal work on AD). Despite this, research on AD in referential communication typically uses visual perspective as its paradigm mental state [4,22,66], while adaptation based on listener-specific traits has gone systematically overlooked. We have designed a task that enables experimentally controlled investigations of this key aspect of AD, by means of modifying the age of the addressees. Although there are countless listener-specific traits according to which listener adaptation takes place, age is arguably one of the most salient. Utterance adaptation based on the age of the addressee is also present in many, if not all, of the world’s languages. Child directed speech is an example of such an adaptation [67,68]. The use of tasks that measure different types of adaptations (e.g. visual perspective, world knowledge) provides the opportunity to understand the potentially divergent developmental trajectories of different sources of adaptation.

Constructing tasks that sufficiently measure adolescents’ pragmatic production has the potential to contribute to the debate regarding pragmatic development and its relation to other aspects of cognition, such as ToM and EF, but also attention and—core—language ability (see §4.1). In this debate, the director task [27] and the multiple features task [69] have been used to argue that EF play a crucial role in facilitating the online usage of common ground [35,70]. This is problematic in at least two respects: consider that (i) the director task has been criticized for tapping domain-general processes, such as selective visual attention [33] and (ii) the multiple features task arguably necessitates the retainment of multiple features (e.g. colour, size and/or number) in memory—something that in itself may put a high demand on cognitive processing. Our AD task was specifically designed to avoid this and is, therefore, an important methodological contribution to the field of experimental AD.

Our AD task was designed to tap the use of common ground information during online communication. Few studies have conducted such online communication tasks in middle childhood and adolescence. For example, in many ToM tasks, participants answer questions regarding the thoughts or feelings of a protagonist (for example, in a film clip or vignette) [71–73]. Our task generated notable variance between age groups and we consider it a suitable measure of perspective taking in language production. The utilization of such tasks to outline the developmental trajectory of pragmatic production is valuable, considering that the ability to use information about others’ perspectives in communicative contexts has been shown to correlate with adolescents’ self-reported peer relations [74,75], which in turn play a deciding role in their social life and psychological well-being [76]. Ultimately, variation in pragmatic processing developmentally could explain adult individual differences in AD, which are large [5].

Why do children entering adolescence not routinely use information about the world knowledge of the interlocutor during online production, even when this information is accessible? From a neurobiological perspective, the functional reorganization and structural changes of brain regions important to communicative perspective taking that take place in adolescence [7,34,65] may be ascribed as a causal explanation. Co-development of these neural changes and functional changes are also likely. Another possible functional explanation of this intriguing pattern is that adult interlocutors have systematically been covering up for this lack of perspective taking, for instance, by making assumptions or requesting clarification (e.g. Is Minecraft the game that mom brought you for Christmas?). Upon entering adolescence, social motivation is increased to succeed in the peer group, and cultural references proliferate into adolescent subcultures, increasing the need to systematically take common ground into account. Conceivably, as an individual approaches adulthood, departures from being a cooperative interlocutor may more often result in conversational breakdown, giving rise to more frequent learning opportunities.

Another perspective on these age-related differences, proposed by Grigoroglou & Papafragou [77], is that some listener-specific adjustments (also termed particular-listener adjustments [78]) may be computationally simpler (e.g. adjustments based on knowledge shared by joint experience, as in classical false belief tasks [16]) and thus emerge earlier than costlier adaptations. Our results and previous findings related to AD development together support this view; while consistent success in false-belief tasks is achieved nearly universally by typically developed 5- to 6-year-olds [10,79,80], 5- to 6-year olds do not consistently provide sufficiently informative expressions in order to disambiguate between competing objects that are visually accessible to the addressee (in 75% of trials) [4]. Building on this perspective, world knowledge-based adjustments may develop even later: 7-year-olds adapted their utterances when the referent was unknown to the addressee in 32% of trials [3], and our findings suggest that this ability continues to successively develop in adolescence.

Which are the underlying factors for these late and successive changes in AD performance? Listener-specific adjustments in production have been described as possibly relying on three critical and interrelated components: (i) ability to attribute knowledge states to others (reflected by our KAS score), (ii) EF (reflected by our WCST scores) and (iii) experience with social interaction [35,40]. Our findings suggest that the development of AD cannot be reduced simply to possessing the relevant information regarding the interlocutor’s knowledge state and high EF. Measuring and testing for the amount of social experience (which can be assumed to increase with age) is of course difficult, but we can turn to what is possibly acquired from experience of social interaction: the ability to use language in specific contexts, in accordance with the social and linguistic conventions of a given community. This conventionalized language use can be regarded as a key feature of pragmatic competence. It has previously been suggested that pragmatic competence should be regarded as a specific capability with its own characterization in terms of developmental trajectory and neurophysiological correlates, rather than a composition of other, more domain-general, skills [5,81]. Our null findings regarding the link between AD and EF speaks in favour of this account.

### 4.1. Limitations

Referential production tasks conducted with passive confederates (as in the current study and [3,5,82]) have been criticized since interactive contexts increase the informativeness of both child and adult speakers’ referential expressions [57]. Motivation to adjust referential expressions to hearers may have been increased with the current paradigm if hearers would have been active conversational partners, potentially even known to the participant. We did, however, conduct the experiment with speaker motivation in mind and, apart from giving a detailed account of the two addressees so that speakers would have a clear representation of them, we presented the task as a challenge in which success depended on whether the addressee would be able to pick the correct referent when listening to the speaker’s directions. Furthermore, it is possible the observed variance between the age groups does reflect a difference in level of automation in the behaviour of adapting utterances to the inferred knowledge of others.

Individual language ability was not included as a variable in our models. Many developmental studies investigating formal language abilities (i.e. vocabulary or morphosyntax) as predictors of pragmatic ability find a medium to strong correlation, although evidence from referential communication is mixed (for a review, see [40]). However, demands on formal language were controlled for in our study; the target and competitor referents in the AD task were carefully chosen, so that a noun phrase with a maximum of one modifier (an adjectival or prepositional phrase), e.g. ‘the blue bird’, would suffice in producing an adequate answer. Such phrases are produced from a very young age (e.g. 2-year-olds in Clark *et al.* [83]), and should, therefore, be easy to produce by 11- to 16-year-old native fluent speakers with no language impairments. Moreover, we believe it is very unlikely that the observed difference in AD is due to differences in verbal IQ. In a previous study conducted by our laboratory, that involved 7-year-olds performing the same AD task, neither of our language ability measures (productive grammar, productive vocabulary) correlated with AD behaviour [3] and the vocabulary task that we conducted (PPVT) is known to be highly correlated with verbal IQ [84]. In summary, the influence of formal language abilities or verbal IQ on the current results are judged to be minimal.

Although the WCST is regarded as an ecologically valid measure of EF [85], there are some uncertainties regarding which specific EF subfunctions the WCST taps. Individual WCST scores do not reflect individual EF subfunctions [86], and the scores used in this study (proportion perseverative errors and proportion non-perseverative errors) have been shown to mainly correlate with working memory and cognitive flexibility in adolescence [48,49]. In consequence, it is possible that some subfunction that was not reflected in the WCST scores, such as inhibition (although correlating with WCST non-perseverative errors in 7-year-olds [49]), played a role in enabling successful AD performance.

### 4.2. Conclusion

We present evidence for age-related differences in the ability to engage in AD between the ages middle childhood (11–12) and middle adolescence (15–16). Children entering adolescence adapted their utterances in accordance with their own assumptions about their addressees’ knowledge states to a notably low extent, although the assumptions as such were accessed upon prompting and corresponded to the assumptions in the older group. This supports an AD model distinguishing between the representation of others’ knowledge as such and the usage of these representations online, where the latter develops later. When it comes to using world knowledge as an information type, our data suggest a developmental timeline spanning adolescence, possibly resulting in adult individual differences. Furthermore, we provide empirical support for the notion that age-related differences in EF do not explain age-related differences in world knowledge-based AD during online production. In summary, our results speak in favour of a continued adolescent development in pragmatic processing, over and above the development of EF.

## Data Availability

All data and code are provided at the following OSF repository: https://osf.io/4y8bs/ [54]. The data are provided in electronic supplementary material [87].

## Footnotes

1 https://www.psytoolkit.org/experiment-library/.
